# Classification of pyroptosis patterns and construction of a novel prognostic model for prostate cancer based on bulk and single-cell RNA sequencing

**DOI:** 10.3389/fendo.2022.1003594

**Published:** 2022-08-29

**Authors:** Jianhan Fu, Guoqiang Li, Ruixiang Luo, Zhijie Lu, Yinhuai Wang

**Affiliations:** Department of Urology, The Second Xiangya Hospital, Central South University, Changsha, China

**Keywords:** prostate cancer, pyroptosis, recurrence-free survival, tumor immune microenvironment, single-cell sequencing

## Abstract

**Background:**

Emerging evidence suggests an important role for pyroptosis in tumorigenesis and recurrence, but it remains to be elucidated in prostate cancer (PCa). Considering the low accuracy of common clinical predictors of PCa recurrence, we aimed to develop a novel pyroptosis-related signature to predict the prognosis of PCa patients based on integrative analyses of bulk and single-cell RNA sequencing (RNA-seq) profiling.

**Methods:**

The RNA-seq data of PCa patients was downloaded from several online databases. PCa patients were stratified into two Classes by unsupervised clustering. A novel signature was constructed by Cox and the Least Absolute Shrinkage and Selection Operator (LASSO) regression. The Kaplan-Meier curve was employed to evaluate the prognostic value of this signature and the single sample Gene Set Enrichment Analysis (ssGSEA) algorithm was used to analysis tumor-infiltrating immune cells. At single-cell level, we also classified the malignant cells into two Classes and constructed cell developmental trajectories and cell-cell interaction networks. Furthermore, RT-qPCR and immunofluorescence were used to validate the expression of core pyroptosis-related genes.

**Results:**

Twelve prognostic pyroptosis-related genes were identified and used to classify PCa patients into two prognostic Classes. We constructed a signature that identified PCa patients with different risks of recurrence and the risk score was proven to be an independent predictor of the recurrence free survival (RFS). Patients in the high-risk group had a significantly lower RFS (P<0.001). The expression of various immune cells differed between the two Classes. At the single-cell level, we classified the malignant cells into two Classes and described the heterogeneity. In addition, we observed that malignant cells may shift from Class1 to Class2 and thus have a worse prognosis.

**Conclusion:**

We have constructed a robust pyroptosis-related signature to predict the RFS of PCa patients and described the heterogeneity of prostate cancer cells in terms of pyroptosis.

## Introduction

Prostate cancer (PCa) is the most common cancer of the genitourinary system among male patients and poses a great burden both on individuals and society. An estimated 1.3 million patients are newly diagnosed with PCa each year (1). The Incidence of PCa vary from 6.3 to 83.4 per 100,000 people across regions. In countries with a high/very high Human Development Index (HDI), the incidence of PCa reached 37.5 per 100,000 people. In countries with a low/median HDI, the incidence of PCa is 11.3 per 100,000 people. The highest rates found in Northern and Western Europe (2). Patients at different PCa stages show large differences in prognosis. For patients with localized PCa, radical prostatectomy is the first-line treatment, while radical radiation therapy is an option for patients with surgical contraindications (3). An international collaborative Randomized Controlled Trial sages suggested no significant difference in the long-term survival between patients treated with these two radical treatments (4). If diagnosed promptly, the 10-year survival rate of localized PCa is approximately 99% (5). Although only 6% patients with PCa experience distant metastasis, the 5-year survival rate of these patients is approximately 30% (6). Foreman et al. predicted that PCa-related mortalities will reach 927,780 in 2040 (7). Therefore, early identification of patients with recurrence is critical to reduce PCa mortality. However, the accuracy of commonly used clinical features such as prostate specific antigen (PSA), Gleason score and TNM staging are not satisfactory in predicting recurrence (8). Exploring an effective and reliable signature for predicting recurrence is thus important to help urologists make clinical decisions.

Pyroptosis, an inflammatory form of programmed cell death (PCD), is a sequence of processes involving the inflammasome, caspase-1, and the gesdermin family (9). The main pyroptosis pathways include the canonical pathway, the non-canonical pathway, the granzyme-mediated pathway, and the caspase-3/8-mediated pathway (10–13). In the canonical pathway, intracellular signaling induced by microbial infection or tumor proliferation, invasion, and metastasis (14) is recognized by pattern recognition receptors (PPRs), resulting in the assembly of the inflammasome (NLRP1, NLRP3, NLRC4, AIM2, and pyrin) and the recruitment of pro-caspase-1 (15). When inflammasome assembly is complete, caspase-1 is activated and cleaves the executor protein gasdermin-D (GSDMD) at Asp275 into two cleavage proteins: N-GSDMD and C-GSDMD. N-GSDMD forms non-selective pores on the cell membrane, causing an unbalanced osmotic potential and leading to water influx and cell swelling (16). Furthermore, IL-1β and IL-18 mature with the help of caspase-1 leak from the N-GSDMD pores, resulting in pyroptosis (17). The mechanisms of other pyroptosis pathways are similar to that of the canonical pathway but involve other inflammasomes, caspase families, and gesdermin families.

Although early studies on pyroptosis focused on the innate immune and inflammatory response to infection, increasing studies have demonstrated that pyroptosis plays a crucial role in cancer, especially in anticancer immunity (18). Jiang et al. verified that miR-21-5p induced pyroptosis which released IL-1β and IL-18 to suppress colorectal cancer growth (19). Another study found that PD-L1 induced pyroptosis in breast cancer, resulting an immune microenvironment unfavorable for tumor growth (20). In the last few years, several drugs and compounds and inorganic selenium have been proven to induce pyroptosis in PCa cells and have antitumor potential (21–23). These findings indicate that pyroptosis may be a potential therapeutic target for PCa patients. However, the relationship between the pyroptosis patterns, immune microenvironment and prognosis in PCa patients remains unknown. Single-cell sequencing (scRNA-seq) uses different types of individual cells as the basic unit of transcriptome analysis, which has unprecedented advantages in studying intra-tumor heterogeneity and tumor immune microenvironment (24). The application of scRNA-seq technology to study cancer cell pyroptosis is promising.

Here, we characterized 2 distinct pyroptosis patterns (Class1 and Class2) based on bulk RNA-seq and scRNA-seq data of PCa patients. On the basis of these patterns, we constructed a prognostic signature and validated the robustness and reliability of this signature. We further investigated the different tumor immune microenvironment between the two Classes. At the single-cell level, we find that the pyroptosis pattern of malignant cells may shift from Class1 to Class2 during the development of prostate cancer. In addition, we constructed the potential ligand-receptor-target networks between malignant cells and other cells.

## Methods

### Data collection

The RNA-sequencing (RNA-seq) data, phenotype data, and survival data of 499 PCa patients and 52 patients with healthy prostates of The Cancer Genome Atlas Prostate adenocarcinoma (TCGA-PRAD) cohort were downloaded from UCSC Xena (http://xena.ucsc.edu/). The RNA-seq data and corresponding clinical features of validation cohorts were downloaded from the Gene Expression Omnibus (GEO) repository (https://www.ncbi.nlm.nih.gov/geo/,GSE157703) (25) and International Cancer Genome Consortium (ICGC) database (https://dcc.icgc.org). RNA-seq data were measured by fragments per kilobase of exon model per million mapped fragments value and normalized before further analyses.

We summarized 55 pyroptosis-related genes from the Molecular Signatures Database v7.2 (https://www.gsea-msigdb.org/gsea/msigdb/index.jsp) (26) and published reviews (27–30). These genes are listed in **Supplementary Table S1**.

### Identification of distinct pyroptosis patterns in PCa patients

To investigate whether pyroptosis-related genes are associated with the prognosis of prostate cancer, we first performed univariate Cox analysis of 55 pyroptosis-related genes with all patients in TCGA-PRAD cohort. Genes with a P<0.05 were considered as prognostic genes. We used the STRING database (version 11.0) (31) to construct a protein-protein interaction (PPI) network for these prognostic genes. To further explore the pyroptosis patterns, the “ConsensusClusterPlus” R package was used to perform a consensus clustering analysis based on the prognostic pyroptosis-related genes.

### Differential expression analysis between different consensus classes

The “DESeq2” R package was used to identify differentially expressed genes (DEGs) between different consensus Classes. Genes with |Log_2_(fold-change) | > 1 and P-adjusted< 0.05 were defined as DEGs and selected for further analysis. On the basis of the expression of DEGs, the “prcomp” function of “stats” R package was used to perform principal component analysis. The “survival” R package was used to perform Kaplan–Meier survival curve analysis and the Log-rank test.

### Functional analyses of the DEGs

The DEGs between distinct consensus Classes were examined by Gene Ontology (GO) and Kyoto Encyclopedia of Genes and Genomes (KEGG) analyses using “clusterProfiler” R package. The Benjamini–Hochberg method was used to adjust P values and P-adjusted<0.05 was selected as the statistical threshold.

### Construction and validation of the prognostic gene signature

We conducted univariate Cox analysis to identify genes with prognostic value by screening the relationship between DEG expression and recurrence-free survival (RFS). To construct an optimal model, the least absolute shrinkage and selection operator (LASSO) penalized Cox regression analysis was used with R package “glmnet”. In this regression, the dependent variables were RFS and the survival status of the patients and the independent variable was the normalized candidate DEG expression. The tenfold cross-validation was used to select the penalty parameter (λ) with the minimum criteria. The risk scoring model was constructed according to each prognostic DEG expression level and its LASSO coefficient. The formula was as follows: risk score $\sum_{i=1}^{n}(Coef_{i}*x_{i}),$ .. = ere *n* ndicates the number of genes in this model, *Coef*_*i*_ s the LASSO coefficient, and *x*_*i*_ the z-score-transformed relative expression value of each selected gene.

We calculated the risk score of each patient in TCGA-PRAD cohort, and patients were divided into high-risk and low-risk groups according to the median value of the risk score. The “survival,” “survminer” and “time-ROC” R packages were used to compare the RFS between the distinct consensus Classes by Kaplan–Meier analysis and generate a receiver operating characteristic (ROC) curve. P<0.05 in two-sided log-rank test was considered significant.

The ICGC-PRAD cohort was analyzed as an external validation cohort. The risk scoring, Kaplan–Meier analysis and ROC curve analysis were performed identically as with the TCGA cohort.

### Independent prognostic analysis of consensus classes and the risk score

We collected the clinical data (age, T stage, Gleason score, and PSA value in TCGA-PRAD cohort) of patients for further analysis. These clinical variables were examined by univariate and multivariate Cox regression analyses along with consensus Classes and the risk score. P<0.05 was considered statistically significant.

### Processing scRNA-seq data

We downloaded the scRNA-seq data of cancerous prostate tissues from GEO database (https://www.ncbi.nlm.nih.gov/geo/,GSE157703) (25). This single-cell transcriptome data was done with 10X Genomics and had been processed into an expression matrix. “Seurat” R package (Version 3.0) was used for subsequent analysis (32). After we employed “harmony” R package to integrate all samples (33), cells with more than 20% expression of mitochondrial genes or fewer than 200 total expressed genes were excluded. We used “NormalizeData” function to eliminate the effect of cell sequencing depths, “FindVariableFeatures” function to find top 2000 feature genes with the highest variance and “ScaleData” function to scale and center features in the dataset. After we conducted “RunPCA” function to reduce the dimension by Principal Component Analysis (PCA), 15 components (PCs) were conserved. “FindNeighbors” and “FindClusters” functions (resolution=0.5) were used to cluster all the cells, while “RunTSNE” and “RunUMAP” functions were used for further dimensionality reduction and visualization of the data. “DoubletFinder” R package was used to detect and remove doublets based on Poisson distribution with an officially recommended DoubletRate(5.4% for 7000 cells) (34). All cells were annotated by “SingleR” R package and Classical cell type markers were checked manually (35). Malignant epithelial cells were identified by “copykat” R package (36) with these parameters: ngene.chr=5, win.size=25, KS.cut=0.1, distance= “Euclidean”.

### Further analysis between the two classes at single-cell level

After we assigned malignant epithelial cells into Class1, Class2 or Not Defined based on the result of “FindAllMarkers” function, “FindMarkers” function was employed to find DEGs between Class1 and Class2 (|Log_2_(fold-change) | > 0.25 and P-adjust< 0.05).

The development trajectories of malignant epithelial cells were constructed by “Monocle” R package (Monocle2) (37). After we inputted the original counts matrix, Monocle2 placed individual cells that are actually in different processes on a same pseudotime corresponding to cell development. Visualization was done by “plot_cell_trajectory” and “plot_pseudotime_heatmap” function.

The differentially enriched pathways between the two Classes were identified by gene set enrichment analysis (GSEA) with P< 0.05 and |normalized enrichment score (NES)| > 0.3. The gene sets were downloaded from the KEGG database. As a method of Over Representation Analysis (ORA) for functional enrichment, “clusterProfiler” R package was also employed with cutoff = 0.5 (38).

Considering a poor prognosis in Class2 patients, cell-cell interactions between Class2 malignant epithelial cells and other cells were analyzed by NicheNet (39). The potential ligand-receptor-target regulatory networks were predicted by calculating DEGs between Class2 and other cells and in combination with known ligand-receptor-target regulatory networks.

### RT-qPCR

RT-qPCR was used to detect the relative mRNA expression of 12 prognostic pyroptosis-related genes: *CASP8, GSDMB, BAK1, BAX, CHMP4B, CHMP4C, CHMP6, TP53, TP63, CASP9, GPX4* and *PLCG1* in RWPE-1 and DU145 cell lines. The total RNA was extracted using TRIzol (THERMO TRIZOL#15596-026) and the cDNA was synthesized using the HiFiScript cDNA Synthesis Kit (CWBIO#CW2569M). The primers were designed by Primer5 software and synthesized by Tsingke Biotechnology (Beijing, China). The primers were shown in **Supplementary Table S2**. The UltraSYBR Mixture (CWBIO#2601) was used as a part of the RT-PCR System. All data were standardized with GAPDH and the relative mRNA expression was calculated by 2^-ΔΔCt^ method.

### Immunofluorescence

The different distribution and expression levels of TP63 in RWPE-1 and DU145 cell lines was detected by immunofluorescence. The cells were fixed by 4% paraformaldehyde for 30 minutes and incubated with primary anti-p63 antibody (1:50, Abcam#ab124762, Cambridge, UK) at 4°C for a night and incubated with CoraLite488-conjugated Affinipure Goat Anti-Rabbit IgG (H+L) (1:100, Proteintech#SA00013-2, Rosemont, USA) at 37°C for 90 minutes. DAPI dye was used to counterstain the cells for 10 minutes. All cells were observed and photographed by microscope (Motic#BA210T).

### Statistical analysis

In our study, the continuous variables were compared by Student’s t-test and the categorical variables were compared by Chi-squared test or Fisher’s exact test. P value adjusted by the Benjamini–Hochberg method was used as the statistical threshold of the GO and KEGG analyses. Kaplan–Meier analysis with a two-side log-rank test was used to compare the RFS between the two groups. Univariable and multivariable Cox regression models was used to assess the prognostic value of the signature. All statistical analyses were performed using R software (v4.1.0) and GraphPad Prism 8. If not specified, P<0.05 was considered statistically significant.

## Resuslts

### Classification of PCa patients according to different pyroptosis patterns

**Figure 1:**
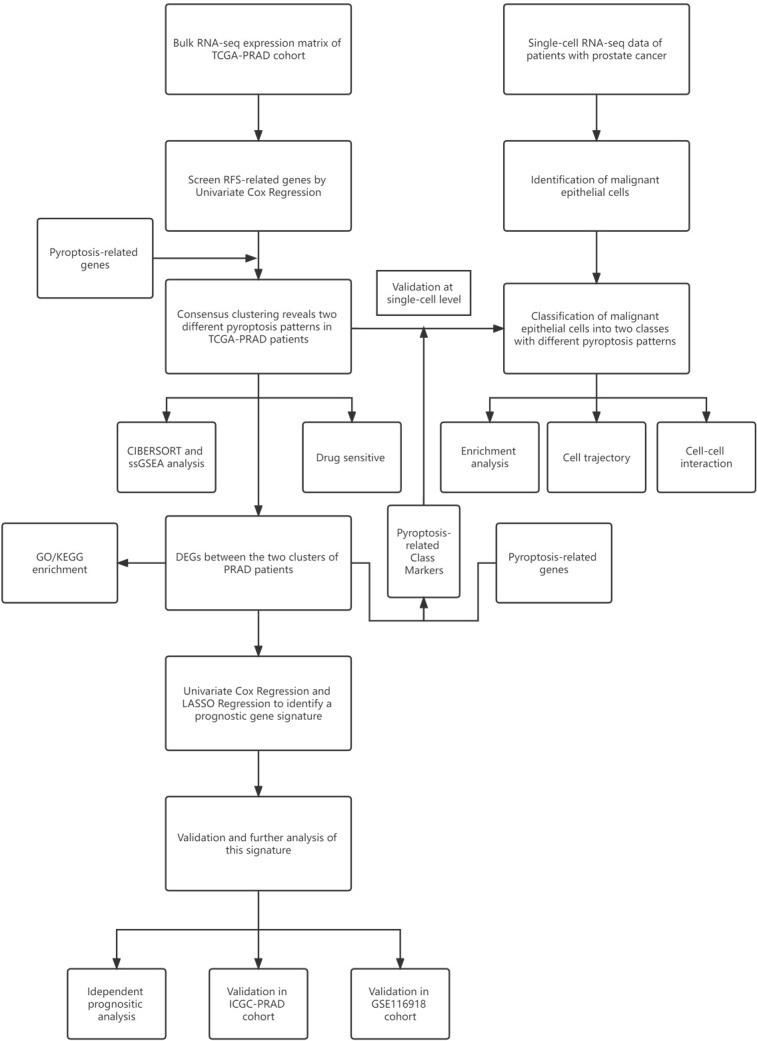
Workflow diagram. The workflow chart of data analysis.

**Figure 2:**
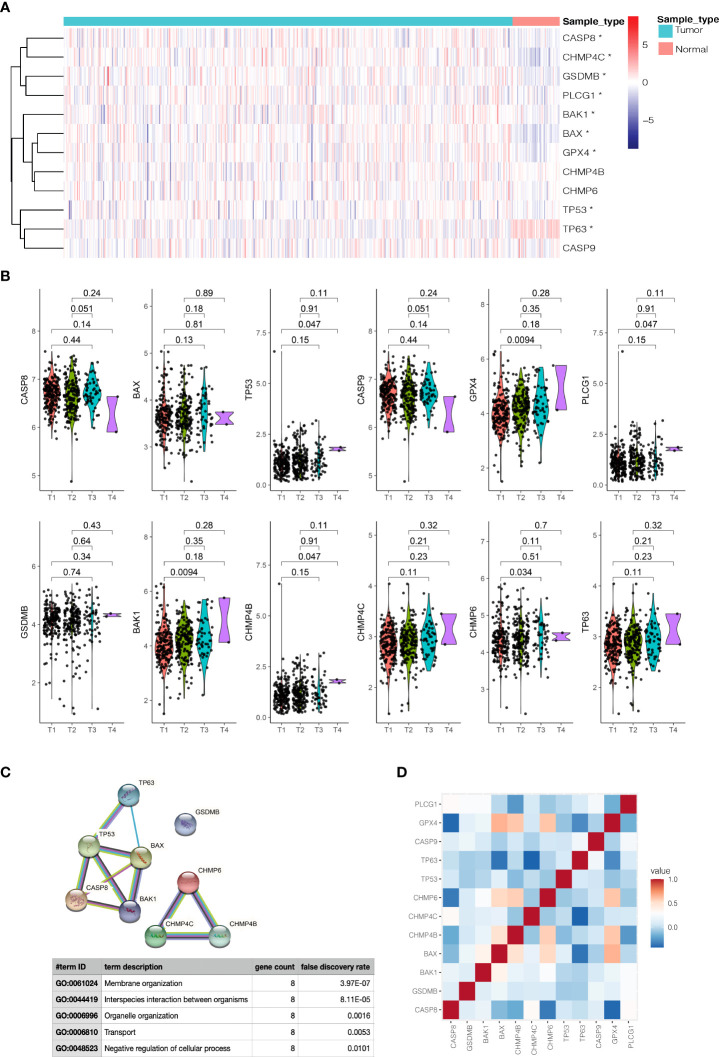
Expressions of 12 prognostic pyroptosis-related genes(PPGs) and clinicopathological features. **(A)** The heatmap(navy:low exression level;red:high expression level) of 12 PPGs between the tumor(blue) and the normal group(pink). P values were shown as: *P<0.05. **(B)** The violin plots of the relationship between 12 PPGs expression and T stage. **(C)** The PPI network generated by STRING database showing the interactions of the pyroptosis-related genes. **(D)** The Pearson correlation heatmap of these prognostic pyroptosis-related genes.

The workflow chart of this study is shown in **Figure 1**. We examined data of 499 PCa patients and 52 patients with healthy prostate in TCGA-PRAD cohort in the preliminary analysis. We performed univariate Cox regression to examine the prognostic value of 55 pyroptosis-related genes in PCa and identified twelve pyroptosis-related genes that predicted RFS: *CASP8, GSDMB, BAK1, BAX, CHMP4B, CHMP4C, CHMP6, TP53, TP63, CASP9, GPX4* and *PLCG1* (P<0.1, **Supplementary Table S3**). RT-qPCR was performed to validate the relative mRNA expression levels of these twelve genes in normal prostate cell line RWPE-1 and prostate cancer cell line DU-145. The results were mostly consistent with the bioinformatic analysis (**Supplementary Figure S1**). The expression of these genes in PCa patients with different clinicopathological characteristics is presented in heatmaps and violin plots (**Figures 2A**, **B**, **Supplementary Figure S2**). The interaction network and Pearson correlation heatmap of these prognostic genes are shown in **Figures 2C**, **D**. We found a high correlation between *BAX* and *GPX4*.

**Figure 3:**
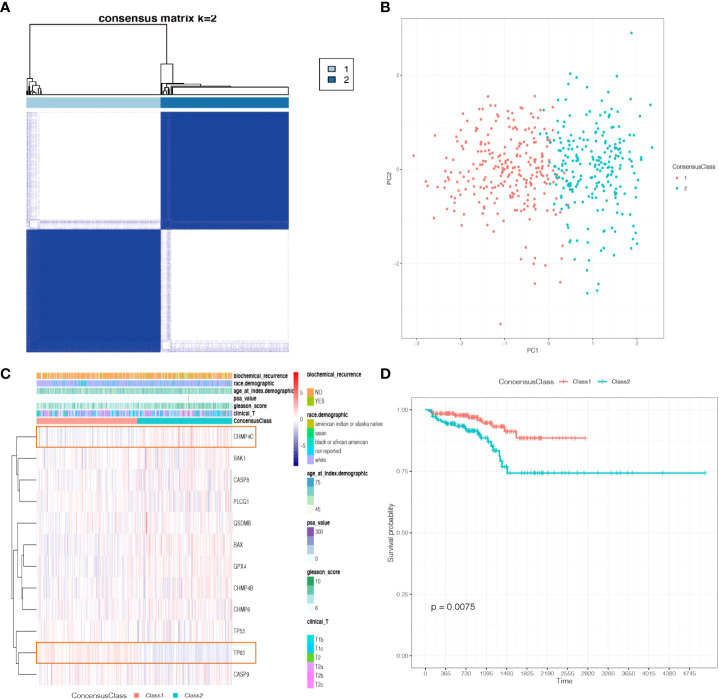
Tumor classification based on the prognostic pyroptosis-related genes. **(A)** 499 PCa patients were divided into two Classes. **(B)** PCA plot based on the gene expression of the two Classes. **(C)** The heatmap and the clinicopathological characteristics of the two Classes. **(D)** Kaplan-Meier RFS curve of the two Classes.

On the basis of the expression data of the twelve prognostic pyroptosis-related genes, we identified distinct pyroptosis patterns using the “ConsensusClusterPlus” R package. We divided the 499 PCa patients into two pyroptosis patterns: 255 cases in consensus Class 1 (Class 1) and 244 cases in consensus Class 2 (Class 2); the correlations between different Consensus Classes were lowest when clustering variable (k) = 2 (**Figure 3A**). The principal components analysis plot indicated that the PCa patients in different Classes were distributed in two directions (**Figure 3B**). The gene expression and clinical features of the two Classes are shown in heatmaps (**Figure 3C**). Class 1 had a significant lower expression of *CHMP4C*, while Class 2 had a significant lower expression of *TP63* (|Log_2_(fold-change) | > 0.5 and P-adjusted< 0.05, **Supplementary Table S6**) and these two genes were defined as Class Markers for further analysis. RFS was compared by Kaplan–Meier curve analysis and log-rank test between the two Classes; Class 1 had a longer RFS compared with Class 2 (P = 0.0075, **Figure 3D**).

### Identification of between the two classes and functional analysis

A total of 1560 genes were identified as DEGs between the two Classes based on the criterion |Log_2_(fold-change) | > 1 and P-adjust< 0.05 (**Supplementary Table S4**). The volcano plot of the DEGs is presented in **Supplementary Figure S2**.

**Figure 4:**
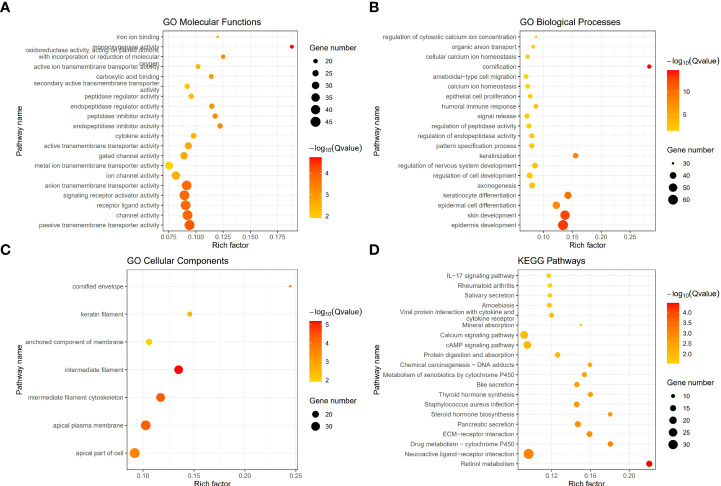
The functional enrichment between the two Classes based on DEGs. **(A)** Barplot graph of the Gene Ontology (GO) Molecular Functions enrichment analysis. **(B)** Barplot graph of the GO Biological Processes enrichment analysis. **(C)** Barplot graph of the GO Cellular Components enrichment analysis. **(D)** Barplot graph of the Kyoto Encyclopedia of Genes and Genomes (KEGG) Pathways enrichment analysis.

To investigate the different functional pathways between the two Classes and explore the potential mechanisms of pyroptosis in PCa, we performed GO and KEGG analyses. The results revealed that some immune-related functional pathways were enriched, such as cytokine activity, humoral immune response and IL-17 signaling pathway. Other signaling pathways were also enriched, such as monooxygenase activity, cornification, cytoskeleton, Calcium signaling pathway and cAMP signaling pathway (**Figures 4A** **–** **D**).

### The distinct tumor immune microenvironment between the two classes

**Figure 5:**
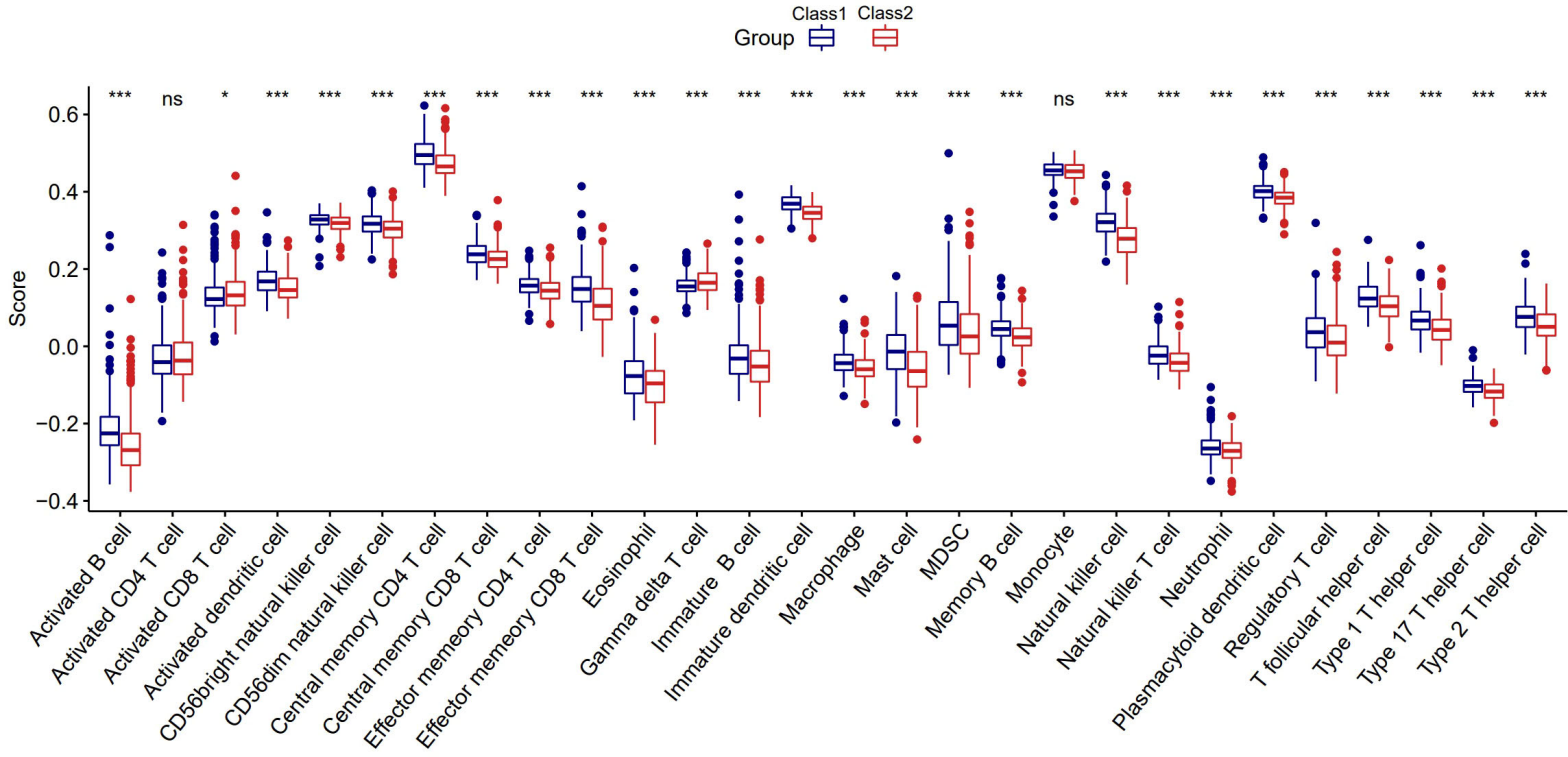
The distinct tumor immune microenvironment between the two Classes. *:p<0.05; ***:p<0.001; ns, not significant.

To further explore whether the impact of the immune system is one of the reasons for the different prognosis of the two Classes, we first used ssGSEA to calculate the enrichment scores of 28 types of immune cells. Surprisingly, the scores of 26 immune cells were significantly different between the two Classes, implying a distinct tumor immune microenvironment (**Figure 5**). We found that most enrichments scores were higher in Class1 than in Class2 (except for activated CD8+ T cells and gamma delta T cells, P<0.05), suggesting that immunosuppression may be responsible for the poor prognosis in Class2 patients.

### The potential therapeutic drugs and diverse sensitivity between the two classes

**Figure 6:**
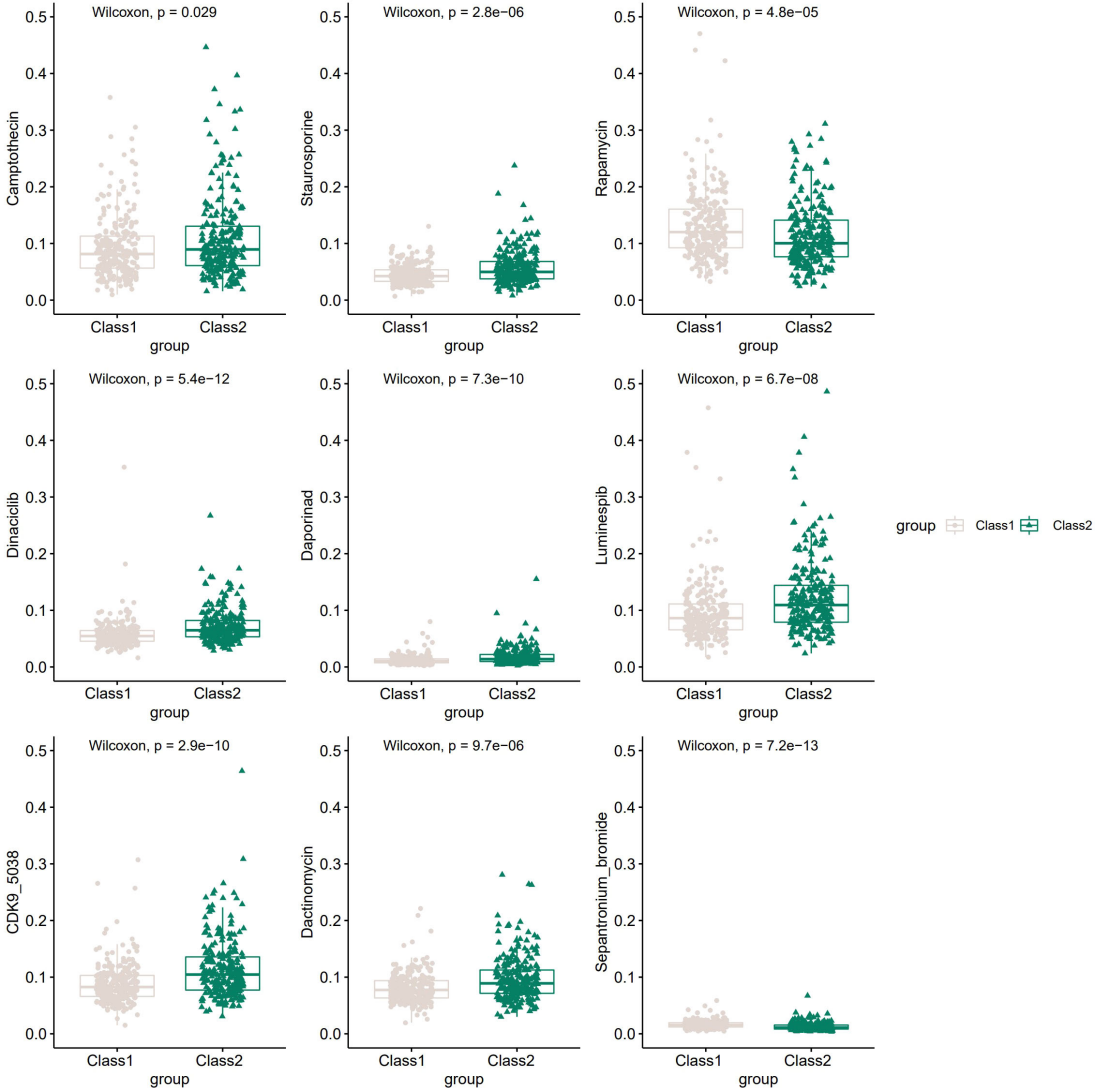
The potential drugs and diverse drug sensitivity between the two Classes of PCa patients.The vertical coordinate represents IC50.

Many compounds have been shown to be potential drugs for prostate cancer, and it is clinically important to study the different sensitivities of these drugs in the two Classes of patients. Hence, we obtained the sensitivity of 481 compounds to 860 cancer cell lines from the Cancer Therapeutic Response Portal (CTRP) database (40–42) and predicted the top 20 sensitive drugs for prostate cancer by “oncoPredict” R package (43). Nine of the top20 compounds showed significant differences in 50%inhibiting concentration (IC50) between the two Classes (**Figure 6**), including Camptothecin, Staurosporine, Rapamycin and many other anti-tumor drugs.

### Construction and validation of a prognostic signature using TCGA-PRAD cohort

**Figure 7:**
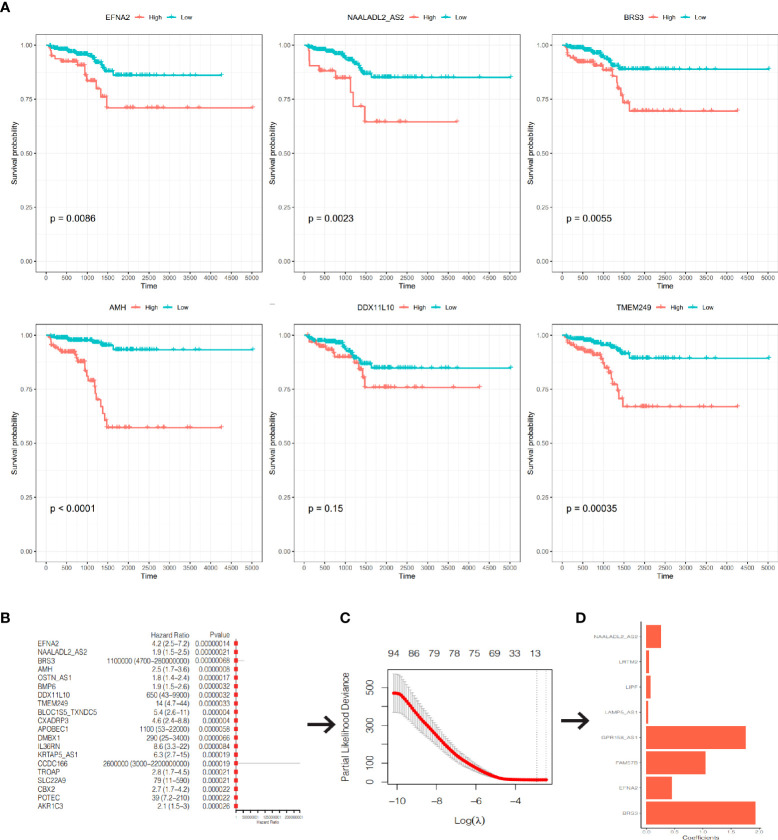
Construction of a prognostic signature. **(A)** The K-M curves of RFS of the top 6 genes with the smallest P-value. **(B)** The forest plot of the top 20 genes with the smallest P-value. **(C)** Partial likelihood deviance was plotted against lag(lambda). The vertical botted lines indicate the lambda value with minimum error. **(D)** The coefficients of the 8 genes in the signature.

After excluding 19 patients without RFS data, 480 PCa patients in TCGA-PRAD cohort were enrolled. We used univariate Cox regression and screened all DEGs; we found that 286 of 1560 DEGs were RFS-related on the basis of the criterion of P< 0.05 **(** **Supplementary Table S4**). The Kaplan–Meier curves of the top six genes with the smallest P-value are presented in **Figure 7A**. A lower expression of *EFNA2*, *NAALADL2_AS2*, *BRS3*, *AMH*, and *TMEM249* were related to a longer RFS.

**Figure 8:**
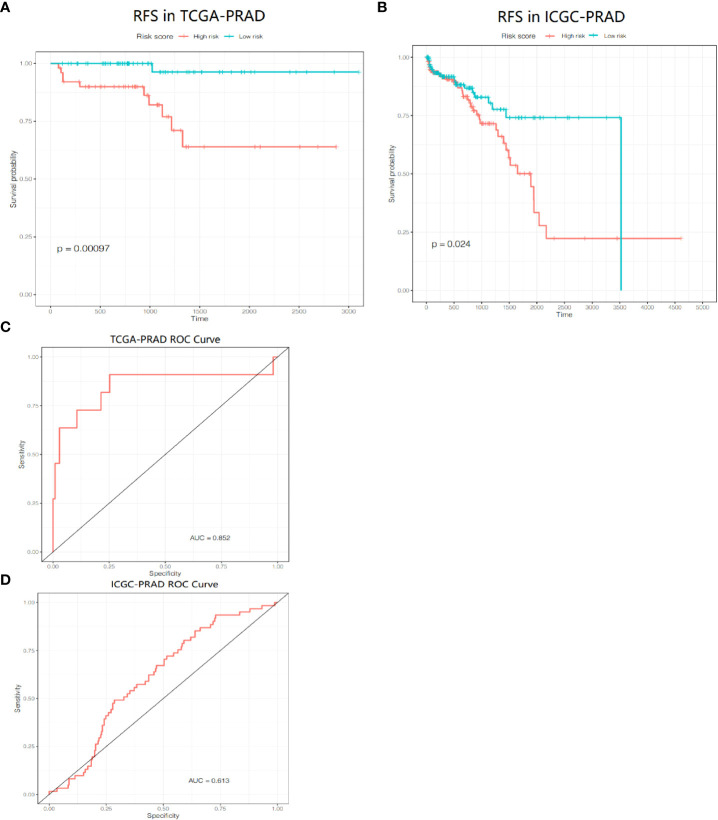
Prognositc value of the gene signature. **(A)** Kaplan-Meier curves for the RFS of patients in the two subgroups in the TCGA-PRAD cohort. **(B)** AUC of ROC curves demonstrated the prognostic value of the risk score in the TCGA-PRAD cohort. **(C)** Kaplan-Meier curves for the RFS of patients in the two subgroups in the ICGC-PRAD cohort. **(D)** AUC of ROC curves demonstrated the prognostic value of the risk score in the ICGC-PRAD cohort.

We included the 286 RFS-related DEGs for LASSO-Cox regression analysis to minimize the risk of overfitting and removed redundant factors (44). Finally, 8 genes were selected to construct a prognostic signature according to the optimal value of λ (**Figures 7B** **–** **D**). The coefficients of these genes are listed in **Supplementary Table S5**. The risk score for each patient was calculated according to the signature gene expression and corresponding coefficient. Since there were overlapping patients in the TCGA-PRAD cohort and the ICGC-PRAD cohort (total overlapping 308 patients), this part of patients was excluded from the survival analysis of the TCGA-PRAD cohort. Using the median value of risk score, the remaining 172 patients in the TCGA-PRAD cohort were equally divided into two groups: the high-risk group and the low-risk group. Patients in the high-risk group had a significantly worse RFS than patients in the low-risk group (**Figure 8A**, P=0.00097). The receiver operating characteristic (ROC) curve showed a very good predictive performance of the prognostic signature with the area under the curve (AUC) of 0.852 (**Figure 8B**).

PCa patients in the ICGC-PRAD cohort were enrolled for further analysis as an external validation to verify the robustness and reliability of this model. These patients were also stratified into two groups based on the median value of the risk score. The probability of tumor recurrence was significantly higher in the high-risk group (**Figure 8C**, P = 0.024). ROC curve showed a good discrimination with an AUC of 0.613 (**Figure 8D**).

### Independent prognostic value of the risk score

**Figure 9:**
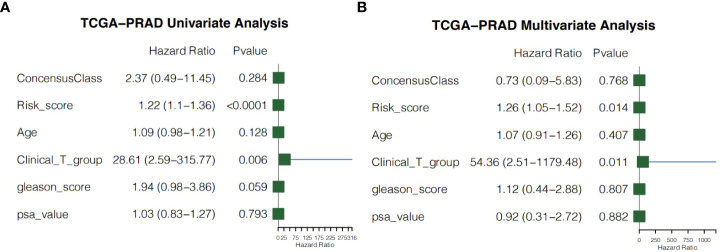
Independent prognostic value of the risk score. **(A)** Univariate cox regression for the TCGA-PRAD cohort. **(B)** Multivariate cox regression for the TCGA-PRAD cohort.

We performed univariate and multivariate Cox regression of other clinicopathological variables in TCGA-PRAD cohort to determine whether the risk score could serve as an independent prognostic factor for RFS in PCa patients. In the univariate Cox regression, the risk score had a higher hazard ratio (HR) and was significantly correlated with poor RFS in both TCGA-PRAD cohort (HR=1.22, 95%CI=1.10–1.36, P<0.0001; **Figure 9A**). While correcting for other confounding clinical factors, multivariate Cox regression indicated that the risk score was still an independent prognostic factor in the TCGA-PRAD cohort (HR=1.26, 95%CI=1.05–1.52, P=0.014; **Figure 9B**).

### Clustering malignant epithelial cells by single-cell analysis

**Figure 10:**
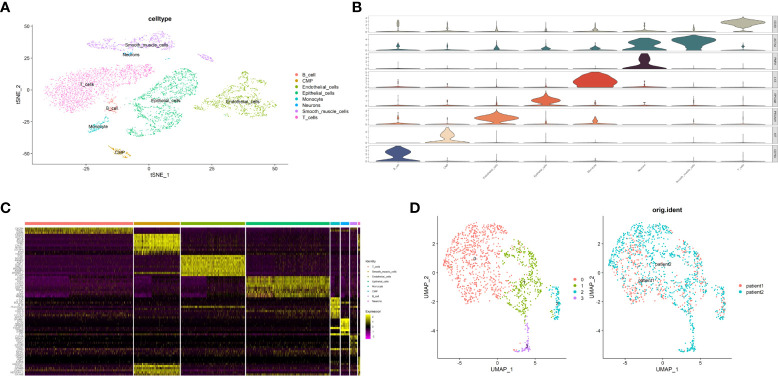
Identification of malignant epithalial cells and subgroups of two PCa patients at single-cell level. **(A)** The t-distributed stochastic neighbor embedding (t-SNE) plot clustering all cells into different clusters. Each dot represents a cell and colored according to its celltype. **(B)** Violin plot demonstrates the accuracy of cell annotation by showing specific markers. **(C)** Heatmap shows the top10 differential expressed genes of each cell type (ordered by average log2|fold change|). **(D)** The uniform manifold approximation and projection (UMAP) plot clustering all malignant epithalial cells into 4 clusters.

To study the different pyroptosis patterns in malignant cells, we analyzed the transcriptome single-cell sequencing data of prostate cancer samples from 2 patients. A total of 6905 cells were obtained after we removed the doublets, and the quality of data was reliable (**Supplementary Figure S3**). We identified 16 clusters of cells (resolution=0.5) and annotated them into 8 distinct cell types (**Figure 10A**), including 1753 epithelial cells, 1351 endothelial cells, 963 smooth muscle cells, 2270 T cells, 150 B cells, 195 monocytes, 180 common myeloid progenitors (CMPs) and 43 neurons. To verified the accuracy of cell annotation, we examined the expression of acknowledged specific markers within each cell type that annotated by “SingleR” R package (**Figures 10B**, **C**). In all epithelial cells, we identified 1258 malignant epithelial cells by “copykat” R package (**Supplementary Figure S4**) and classified them into 4 clusters (**Figure 10D**, resolution=0.2).

### Characterization of malignant epithelial cells based on pyroptosis-related class Markers

**Figure 11:**
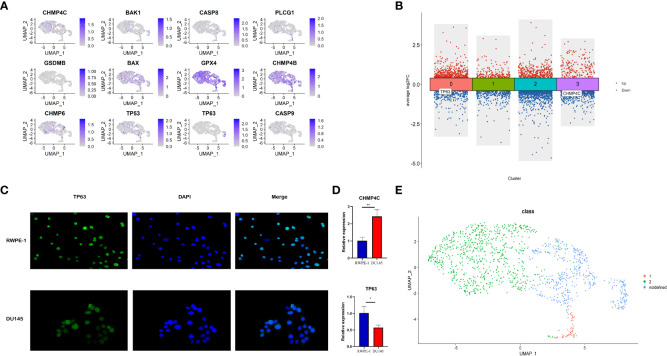
Identification of different pyroptosis patterns at single-cell level according to the expression of class markers (TP63 and CHMP4C). **(A)** Feature plot shows the expressions of 12 PPGs in malignant epithalial cells. **(B)** The DEGs of the 4 clusters of malignant epithelial cells. TP63 was down-regulated in cluster0 and CHMP4C was down-regulated in cluster3. **(C)** The distribution and expression of TP63 in normal prostate cell line RWPE-1 and prostate cancer cell line DU-145. **(D)** The relative mRNA expression level of CHMP4C and TP63 in normal prostate cell line RWPE-1 and prostate cancer cell line DU-145. **(E)** Re-annotation of all malignant epithelial cells into two different pyroptosis patterns. *:p<0.05; **:p<0.01.

The relative expression level of 12 prognostic pyroptosis-related genes (PPGs) was shown in **Figure 11A**. To investigate the pyroptosis pattern in prostate cancer at the single-cell level, we used previously defined Class Markers (CHMP4C and TP63) to re-assign each malignant epithelial cell to Class1, Class2 or Not Defined. The “FindAllMarkers” function of “Seurat” R package was conducted to find the DEGs for each cluster (P< 0.01, **Supplementary Table S7**). The volcano plot shows that CHMP4C was significantly down-regulated in cluster3, and TP63 was significantly down-regulated in cluster0 (**Figure 11B**). To validate these two classifier genes in human cell lines, we studied the distribution and expression of TP63 and the relative mRNA expression level of CHMP4C and TP63 in normal prostate cell line RWPE-1 and prostate cancer cell line DU-145 (**Figures 11C**, **D** **)**. TP63 has a lower expression in prostate cancer cells compared to normal prostate cells, indicating that it may serve as a key molecule in fight against prostate cancer. According to this result, we assigned malignant epithelial cells in cluster 3 as Class1, malignant epithelial cells in cluster 0 as Class2, other malignant epithelial cells as Not Defined (**Figure 11E**).

### Pyroptosis pattern may shifts from class1 to class2

**Figure 12:**
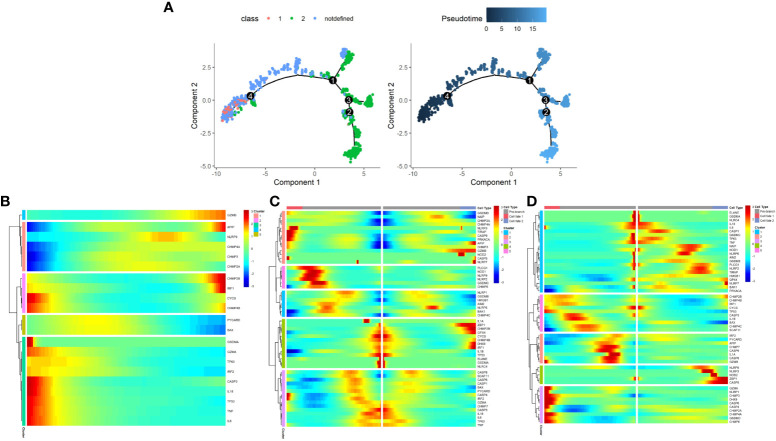
The development trajectory and dynamic change of two Classes of maligant epithelial cells. **(A)** Pseudotime analysis reveals the development trajectories of the two Class of maligant epithelial cells (From Class1 to Class2). **(B)** The dynamic change of the expression of pyroptosis-related genes during the development of maligant epithelial cells (Timeline from left to right). **(C)** Heatmap shows pyroptosis-related genes involved in the differentiation of maligant epithelial cells (branch point1). **(D)** Heatmap shows pyroptosis-related genes involved in the differentiation of maligant epithelial cells (branch point4).

The development of malignant epithelial cells is a dynamic process. We used Monocle 2 algorithm to speculate on the possible developmental trajectory of malignant epithelial cells, and found that the trajectory began with Class1 malignant cells and ended with Class2 malignant cells (**Figure 12A**). We therefore propose the hypothesis that in the development of prostate cancer, the pyroptosis pattern may shifts from Class1 to Class2 and as a result, a poor prognosis (see **Figure 3D**).

In addition, we found a dynamic change in the expression of pyroptosis-related genes during the development of malignant epithelial cells (**Figures 12B**–**D**). For example, GZMB expression increases with the development trajectory while GZMA expression decreases.

### Enrichment analysis between the two classes of malignant epithelial cells

**Figure 13:**
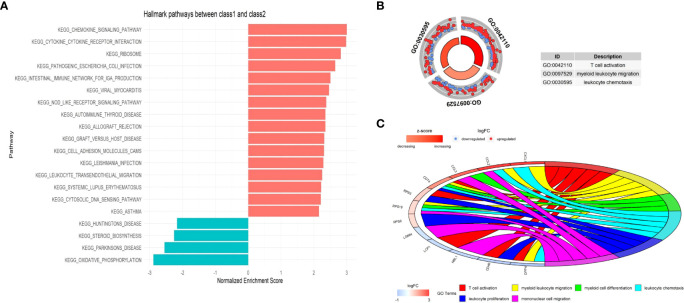
The functional enrichment between the two Classes at single-cell level. **(A)** Immune-related pathways were enriched by Gene Set Enrichment Analysis (GSEA). **(B)** Circle plot presents GO Biological Processes. **(C)** Chord plot presents GO Biological Processes and involved genes.

The different cellular behavior and biological process between the two Classes of malignant epithelial cells could affect the tumor immune microenvironment. Based on the DEGs between Class1 and Class2, several immune-related pathways were enriched by Gene Set Enrichment Analysis (GSEA), such as chemokine signaling and cytokine receptor interaction (**Figure 13A**). GO analysis also indicated a distinct tumor immune microenvironment between the two Classes of malignant cells (**Figures 13B**, **C**).

### The potential cell-cell interaction between malignant epithelial cells and other cells

The “nichenetr” R package was used to predict the potential ligand-receptor pairs and ligand-target gene pairs in the process of malignant cells developing.

**Figure 14:**
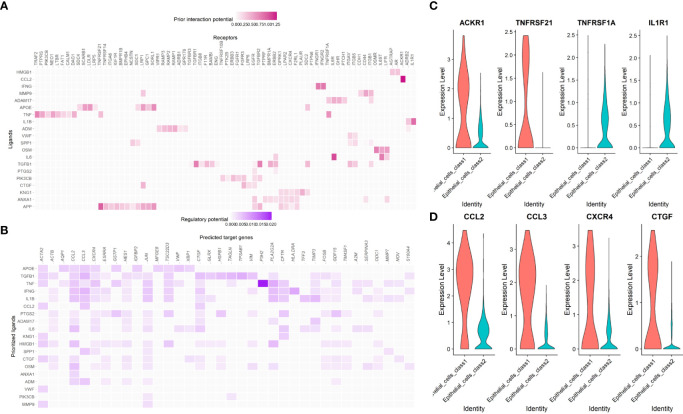
The cell-cell interaction between malignant epithelial cells and other cells. **(A)** The regulatory potential of the prioritized cell ligard-receptor pairs in the development of maligant epithelial cells. **(B)** The regulatory potential of the prioritized cell ligard-target pairs in the development of maligant epithelial cells. **(C)** The expression of the prioritized receptors in the development of maligant epithelial cells. **(D)** The expression of the prioritized targets in the development of maligant epithelial cells.

CCL2-ACKR1, APP- TNFRSF21, IL6-TNFRSF1A and IL1B-IL1R1 were ligand-receptor pairs with strong regulatory potential (**Figure 14A**), while CCL2, CCL3, CXCR4 and CTGF were target genes that regulated by multiple pathways (**Figure 14B**). Similar to the enrichment analysis, this result demonstrates that many immune system components are involved in the shift from Class1 malignant cells to Class2 malignant cells. The expression levels of these receptors and genes also differed between the two Classes (**Figures 14C**, **D**).

## Discussion

PCa is a heterogeneous disease not only in terms of clinical features but also in terms of biological process, and the optimal treatment differs from patient to patient depending on the stage of the disease (45). Therefore, to improve the prognosis of prostate cancer, all patients should undergo regular follow-up to monitor the stage of the disease. However, the commonly used three-tiered system for risk stratification (low-, intermediate- and high-risk of recurrence) does not have a satisfactory efficacy and reliability (46). A novel model to predict the recurrence of PCa is of great importance.

Pyroptosis is a novel type of PCD, and many studies have shown that pyroptosis has potential antitumor functions, especially by affecting the tumor immune microenvironment (19–23, 47). However, other studies showed that pyroptosis can promote the development of cancer (48). The relationship between pyroptosis and the prognosis of PCa is not clear. Considering that PCa is accompanied by mutations that may affect the process of pyroptosis and thus tumor development (49), we aimed to identify different pyroptosis patterns in PCa samples and construct a robust signature for predicting recurrence.

We screened pyroptosis-related genes in PCa samples and Classified PCa patients into two Classes based on twelve prognostic pyroptosis-related genes, with different RFS. We further analyzed the DEGs between the two Classes and constructed a prognostic gene signature. Two external databases were used for validation. We demonstrated a different immune microenvironment between the two Classes at the single-cell level. Importantly, the pyroptosis pattern may shifts from Class1 to Class2, which has a worse prognosis.

In our study, twelve pyroptosis-related genes with prognostic values were identified by univariate Cox regression and a PPI network was constructed. Caspase-8 (encoded by *CASP8*) is a member of the cysteine-aspartic acid protease (caspase) family and plays a key role in PCD by switching apoptosis, necroptosis and pyroptosis (50). Active caspase-8 induces GSDMD-mediated pyroptosis by cleaving GSDMD at the same site as caspase-1 (51). In esophageal squamous cell carcinoma, photodynamic therapy induces pyroptosis by activating caspase-8, indicating caspase-8 as a new target for esophageal squamous cell carcinoma (52). In our study, we found a higher CASP8 expression in PCa patients at T3–T4 staging or with a higher Gleason score or a higher PSA value, suggesting suppression of CASP8 may improve PCa prognosis. BCL2 associated X (BAX), a Classic apoptosis regulator, has been reported to mediate pyroptosis by binding the mitochondrial outer membrane to execute apoptosis (53). A study in 2021 demonstrated that BAX may be a novel target for chemotherapy in colorectal cancer (54). Our results showed that the HR of BAX was 2.8 and that chemotherapy targeting BAX have the potential to be used in PCa patients. BCL2 Antagonist Killer 1 (BAK1) also belongs to the BCL2 protein family. similar to BAX, BAK1 has the ability to switch apoptosis to pyroptosis (55) and can interact with the Tumor protein 53 (TP53) in response to cell stress (56). TP53 is a tumor suppressor protein that regulates a variety of biological processes such as apoptosis, cell cycle, and DNA repair (57). In non-small-cell lung cancer, p53-induced pyroptosis significantly suppressed tumor cell proliferation and recurrence (58). While Tumor protein 63 (TP63) encodes a transcription factor of TP53 and plays a crucial roles in tumorigenesis suppression (59), the HR of TP53 and TP63 was 0.57 and 0.69, respectively, consistent with previous studies. When the immune system is activated, natural killer (NK) cells and cytotoxic T lymphocytes release granzyme A (GzmA) to cleave Gasdermin-B (GSDMB), which is a pore-forming protein that can trigger pyroptosis (60). Our research showed that GSDMB expression increased markedly in the PCa patients, indicating that GSDMB-dependent pyroptosis may be one of the responses of the immune system to the tumorigenesis of PCa. Glutathione peroxidase 4 (GPX4) protects cells from oxidative damage by catalyzing the reduction of organic hydroperoxides. Kang et al. found that the knockout of GPX4 in myeloid lineage cells induces caspase-11-dependent GSDMD cleavage and triggers macrophage pyroptosis in the PLCG1-dependent pathway (61). The GO analysis enriched in monooxygenase activity, suggesting that GPX4 may affect the pyroptosis by activating enzymes related to oxidation. Charged Multivesicular Body Protein (CHMP) family, including *CHMP4B, CHMP4C* and *CHMP6*, are part of the endosomal sorting complex required for transport (ESCRT). A study published in 2020 shows that after pyroptosis, an ESCRT-mediated plasma membrane repair also occurs to avoid cell death (62). In the current study, a higher expression of CHMP4B, CHMP4C or CHMP6 increased the risk of recurrence in PCa patients, suggesting that they may assist PCa cells in escaping pyroptosis. Inhibitors of CHMP family may be one of the possible therapeutic targets for PCa patients. However, the specific molecular mechanisms by which the proteins encoded by these pyroptosis-related genes affect the prognosis of PCa should be further studied.

Several studies showed that genes associated with PCD can predict the prognosis of PCa patients. An apoptosis-related gene signature and ferroptosis-related gene signature had a prognostic value in predicting RFS for PCa patients with an AUC of 0.787 and 0.767, respectively (63, 64). The relationship between different pyroptosis patterns and PCa prognosis remains unclear. On the basis of the two Classes of PCa patients identified by consensus clustering, we constructed a pyroptosis-related prognostic model with an AUC of 0.852, which had a better predictive performance compared with that of other PCD prognostic models. PCa patients were distributed into the high-risk group and the low-risk group according to the median value of the risk score. We performed an external validation using the ICGC-PRAD database.

The result of functional enrichment analysis at both bulk RNA-seq level and single-cell level indicated the presence of differences in the tumor immune microenvironment between the two Classes, such as cytokine pathway, humoral immune response and IL-17 signaling pathway. Programmed cell death ligand 1 (PD-L1) inhibitors kill tumor cells by triggering pyroptosis (65), while IL-17 promotes PD-L1 expression in tumor cells by PD-1+ immune cell intratumor infiltration (66), suggesting that IL-17 may be a potential target for enhancing the performance of PD-L1 inhibitors by increasing pyroptosis of tumor cells. We observed a higher infiltration of macrophages in Class1, while it has been reported that macrophages can release reactive oxygen species (ROS) and thus trigger pyroptosis (67). The oxidation-related gene GPX4 could affect the prognosis of PCa patients, suggesting the redox response may have an interplay with pyroptosis in PCa. In addition, we found that TNF is a ligand with high regulatory potential in the development of malignant cells, and it is mainly secreted by macrophages (68). The interplay between the cancer cell pyroptosis and macrophages has yet to be elucidated.

This study has several limitations. First, our data were collected from online databases such as TCGA and GEO. Patients in public databases are heterogeneous in clinical features such as treatments, Gleason score, and stage, which may impair the accuracy of the signature. More real-world data, especially ethnically diverse populations and metastatic prostate cancer that collected prospectively with rigorous patient stratification are needed to further validate the prognostic value of this model. Second, experiments are needed to demonstrate the specific mechanism of pyroptosis in PCa, especially how it affects the immune microenvironment. Third, although the 10X Genomic platform has been widely used for single-cell sequencing, it does not yield the full-length transcripts and may lose some information. In addition, this model was based only on different patterns of pyroptosis and excluded other factors that may affect the prognosis of PCa.

## Conclusion

We identified two pyroptosis patterns based on the prognostic pyroptosis genes at both bulk RNA-seq level and single-cell level. The DEGs between these two Classes were screened and we constructed a novel prognostic gene signature. This signature was validated by two external databases and was proven to be an independent factor correlated with RFS of PCa patients. Patients in the different Classes had a different immune microenvironment. The technology of single-cell sequencing offers a new perspective for understanding pyroptosis.

## Data availability statement

The original contributions presented in the study are included in the article/supplementary material. Further inquiries can be directed to the corresponding author.

## Ethics statement

Ethical review and approval were not required for the study on human participants in accordance with the local legislation and institutional requirements. Written informed consent for participation was not required for this study in accordance with the national legislation and the institutional requirements.

## Author contributions

YW contributed to the conception of the study; JF and GL performed the bioinformatic analysis; JF visualized the data and wrote the manuscript; RL and ZL helped perform the analysis with constructive discussions. All authors contributed to the article and approved the submitted version.

## Acknowledgement

We thank Gabrielle White Wolf, PhD, from Liwen Bianji (Edanz) (www.liwenbianji.cn/), for editing the English text of a draft of this manuscript.

## Conflict of interest

The authors declare that the research was conducted in the absence of any commercial or financial relationships that could be construed as a potential conflict of interest.

## Publisher’s note

All claims expressed in this article are solely those of the authors and do not necessarily represent those of their affiliated organizations, or those of the publisher, the editors and the reviewers. Any product that may be evaluated in this article, or claim that may be made by its manufacturer, is not guaranteed or endorsed by the publisher.

## Supplementary material

Supplementary Tables 1 The pyroptosis-related genes.

Supplementary Tables 2 The primers of RT-qPCR.

Supplementary Tables 3 The prognostic value of pyroptosis-related genes.

Supplementary Tables 4 The DEGs between the two Classes.

Supplementary Tables 5 The coefficients of the 8 genes in the signature.

Supplementary Tables 6 The different expression pattern of 12 PPGs between the two Classes.

Supplementary Tables 7 The DEGs between the 4 clusters of malignant epithelial cells.

Supplementary Figures 1 The relative mRNA expression levels of these twelve genes in normal prostate cell line RWPE-1 and prostate cancer cell line DU-145.

Supplementary Figures 2 The heatmaps(navy:low exression level;red:high expression level) of the relationship between 12 PPGs expression and (C) gleason score, (D) psa value, (E) age, (F) T stage, (G) N stage, (H) M stage.

Supplementary Figures 3 The volcano plot of 1560 DEGs (green: up-regulated in Class 1; red: down-regulated in Class 1).

Supplementary Figures 4 Vlnplot showing the good quality of this public single-cell sequencing data.

Supplementary Figures 5 Using Copykat to identify malignant epithelial cells.

Supplementary Figuresa 6 Dotplot of GO enrichment analysis.
