# Supplementary material for: Classification of pyroptosis patterns and construction of a novel prognostic model for prostate cancer based on bulk and single-cell RNA sequencing
Source: Front Endocrinol (Lausanne). 2022 Aug 29;13:1003594. doi: 10.3389/fendo.2022.1003594 (PMC9465051; doi:10.3389/fendo.2022.1003594)
Supplement: Supplementary Tables 1 — The pyroptosis-related genes. [file DataSheet_1.zip › Supplementary TableS2.docx]

H-actin

<https://www.ncbi.nlm.nih.gov/gene/60>

F ACCCTGAAGTACCCCATCGAG

R AGCACAGCCTGGATAGCAAC

Product length 224bp

1. CASP8
   <https://www.ncbi.nlm.nih.gov/gene/841>
   F GGTAATGACAATCTCGGACTCT
   R AGTATCCCCGAGGTTTGCTT
   Product length 95bp

   H-Bax
   <https://www.ncbi.nlm.nih.gov/gene/581>
   F TTCTGACGGCAACTTCAACTGG
   R TGATCAGTTCCGGCACCTT
   Product length101bp

   H-GPX4
   <https://www.ncbi.nlm.nih.gov/gene/2879>
   F CTTCGTGTGCATCGTCACC
   R AAACCACACTCAGCGTATCGG
   Product length 102bp

   H-CHMP4C
   <https://www.ncbi.nlm.nih.gov/gene/92421>
   F AGCAAAAGCGATGAAATCTGT
   R GTCATCACCAAAGCCAACCC
   Product length 139bp

   H-GSDMB
   <https://www.ncbi.nlm.nih.gov/gene/55876>
   F GGGCCATCTCAGCTATAAACACA
   R GCACCATCCTTCTTCATCGTCT
   Product length 123bp

   H-PLCG1
   <https://www.ncbi.nlm.nih.gov/gene/5335>
   F TTTGCCTTTCTGCGCTTCGT
   R AAGGCACTGCTCTGTATCCTG
   Product length 115bp

   H-BAK1
   <https://www.ncbi.nlm.nih.gov/gene/578>
   F GACGACATCAACCGACGCTA
   R CCCCAATTGATGCCACTCTCA
   Product length 131bp

H-CHMP4B
<https://www.ncbi.nlm.nih.gov/gene/128866>
F TGCTGGAAATCAGTGGACCC
R CGGGTTTTGATGGTAGGGCT
Product length 72bp

H-CHMP6
<https://www.ncbi.nlm.nih.gov/gene/79643>
F ATCCCAGAAAACGTCCCTGT
R GTTCAGGCTCAAGGTCTCCAG
Product length 250bp


H-P53
<https://www.ncbi.nlm.nih.gov/gene/7157>
F GCCCATCCTCACCATCATCACA
R CAGGCACAAACACGCACCTC
Product length 84bp

H-TP63
<https://www.ncbi.nlm.nih.gov/gene/8626>
F CTGCCCTGACCCTTACATCC
R TGGGACATGGTGGATCGGTA
Product length 87bp

H-CASP9
<https://www.ncbi.nlm.nih.gov/gene/842>
F CCAACCCTAGAAAACCTTACCCC
R TGTTTCCGGTCTGAGAACCT
Product length 75bp
