# Supplementary figures and images for: Classification of pyroptosis patterns and construction of a novel prognostic model for prostate cancer based on bulk and single-cell RNA sequencing
Source: Front Endocrinol (Lausanne). 2022 Aug 29;13:1003594. doi: 10.3389/fendo.2022.1003594 (PMC9465051; doi:10.3389/fendo.2022.1003594)

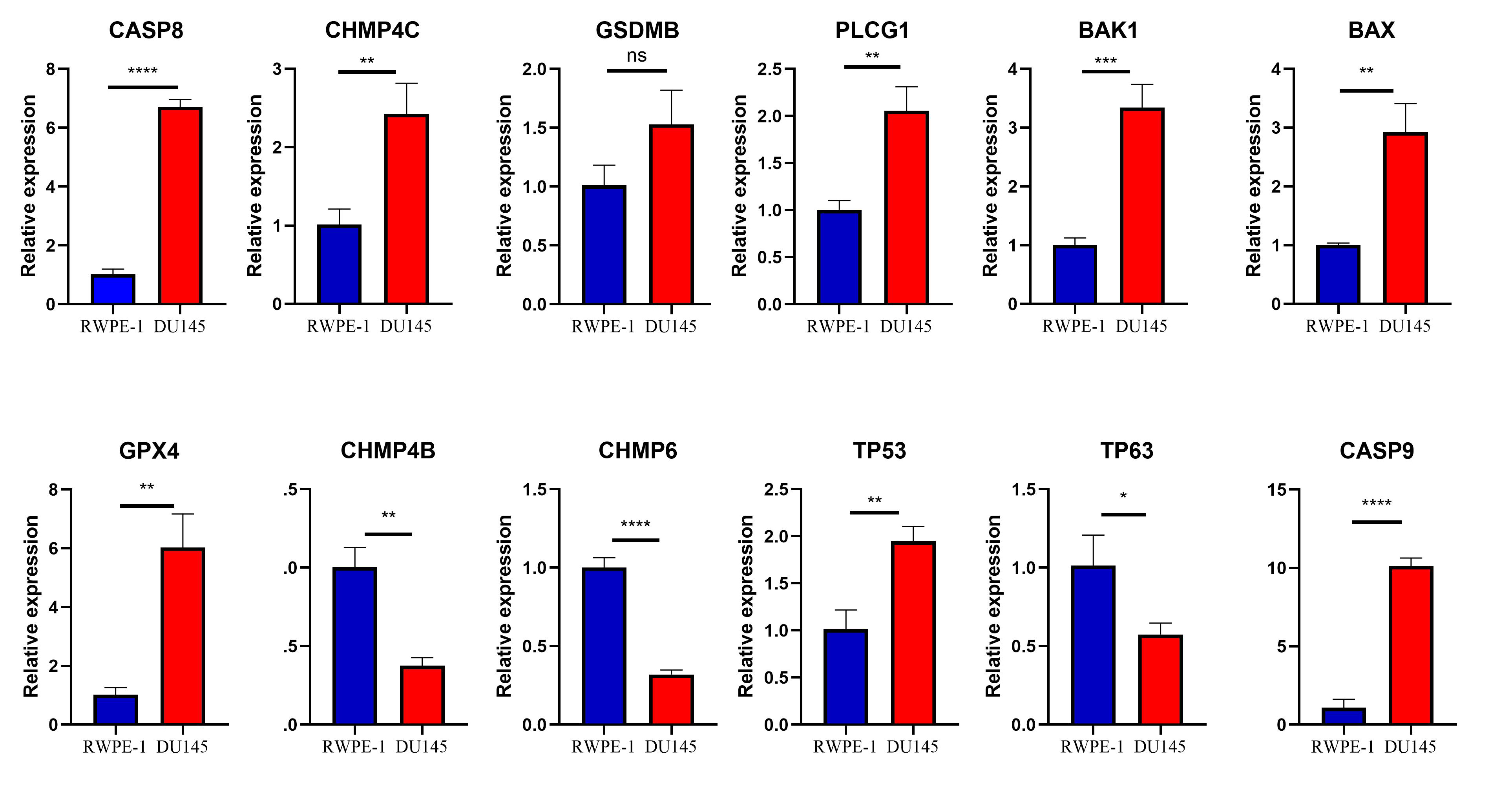

Supplement: Supplementary Tables 1 — The pyroptosis-related genes. [file DataSheet_1.zip › Supplementary FigureS1.jpg]

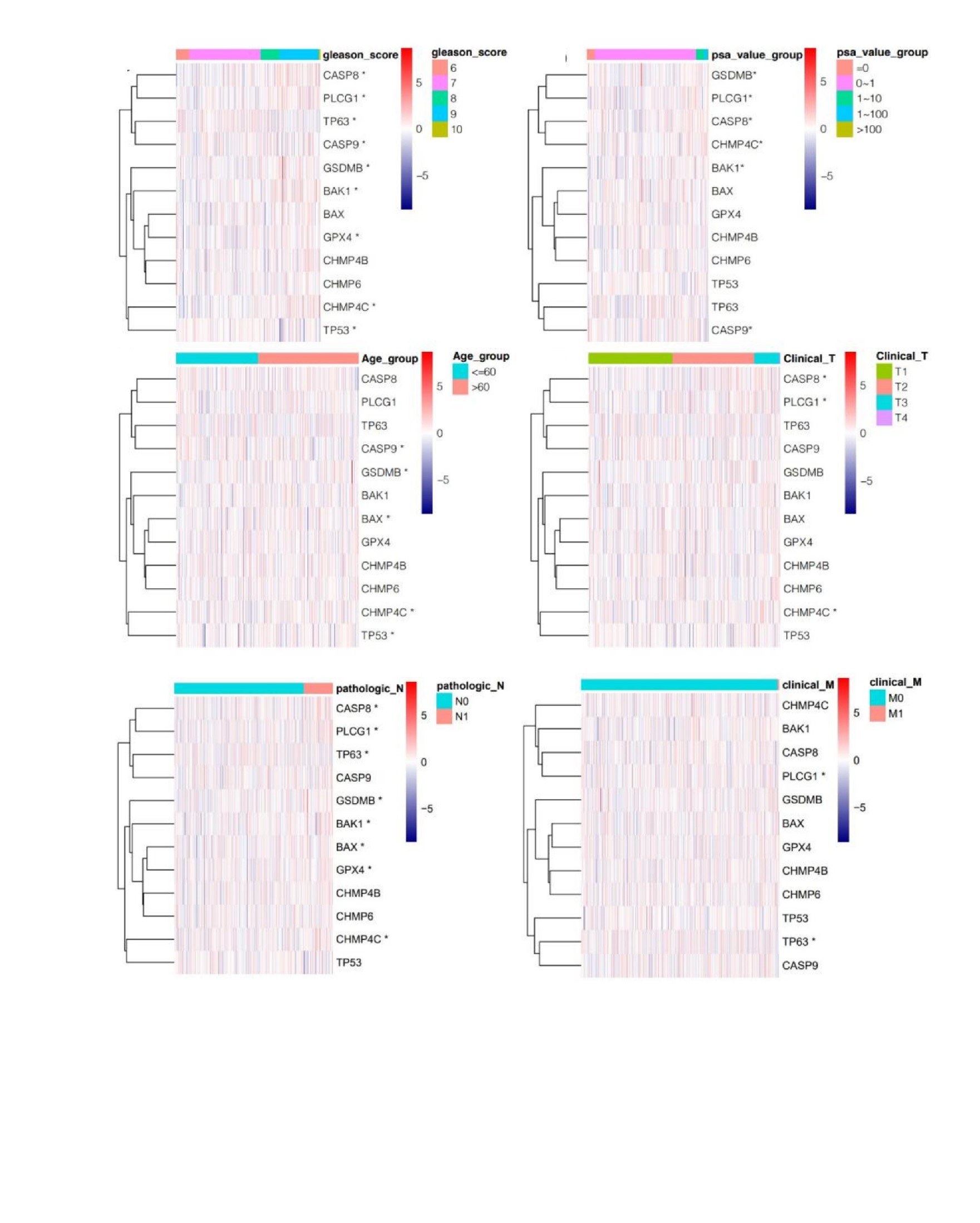

Supplement: Supplementary Tables 1 — The pyroptosis-related genes. [file DataSheet_1.zip › Supplementary FigureS2.jpg]

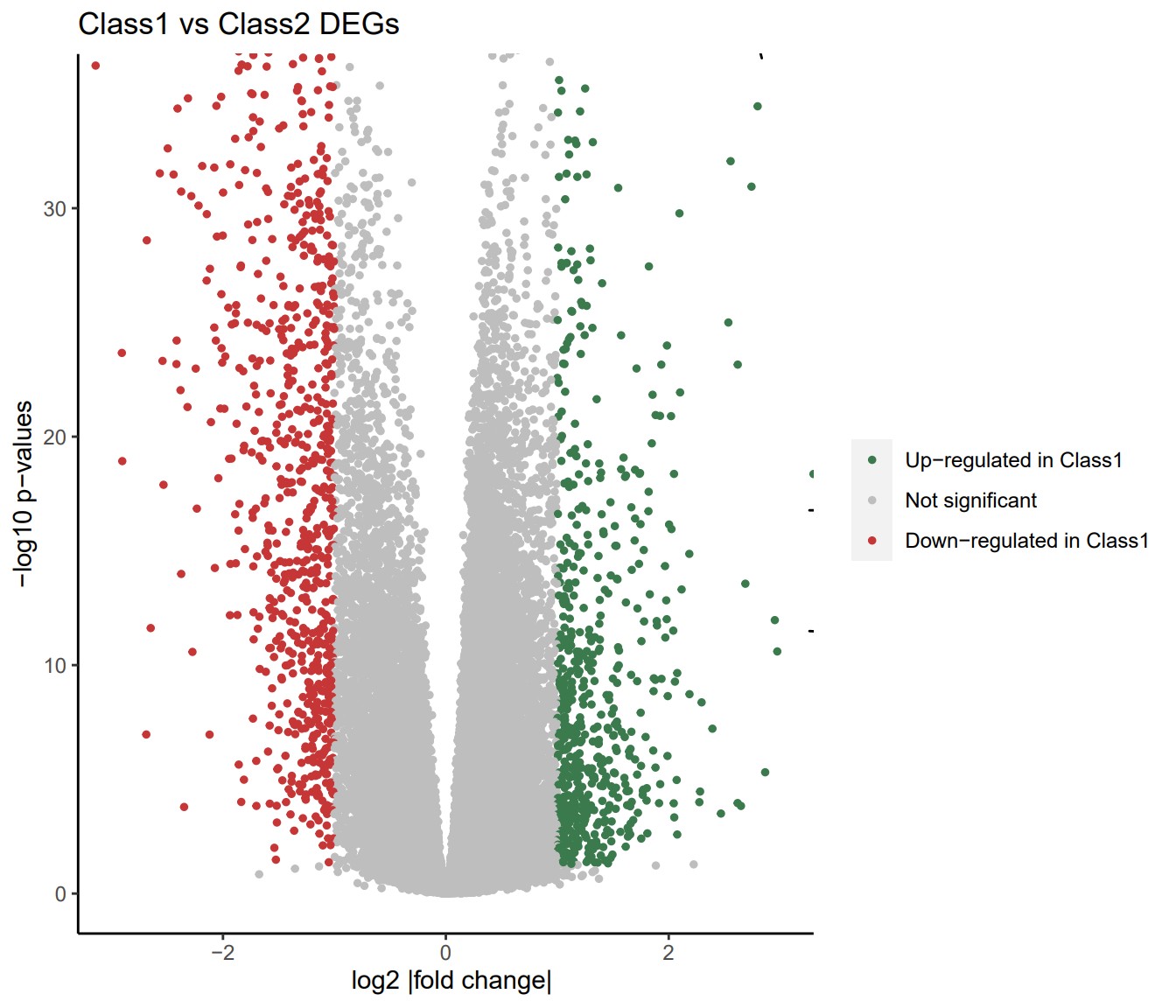

Supplement: Supplementary Tables 1 — The pyroptosis-related genes. [file DataSheet_1.zip › Supplementary FigureS3.jpg]

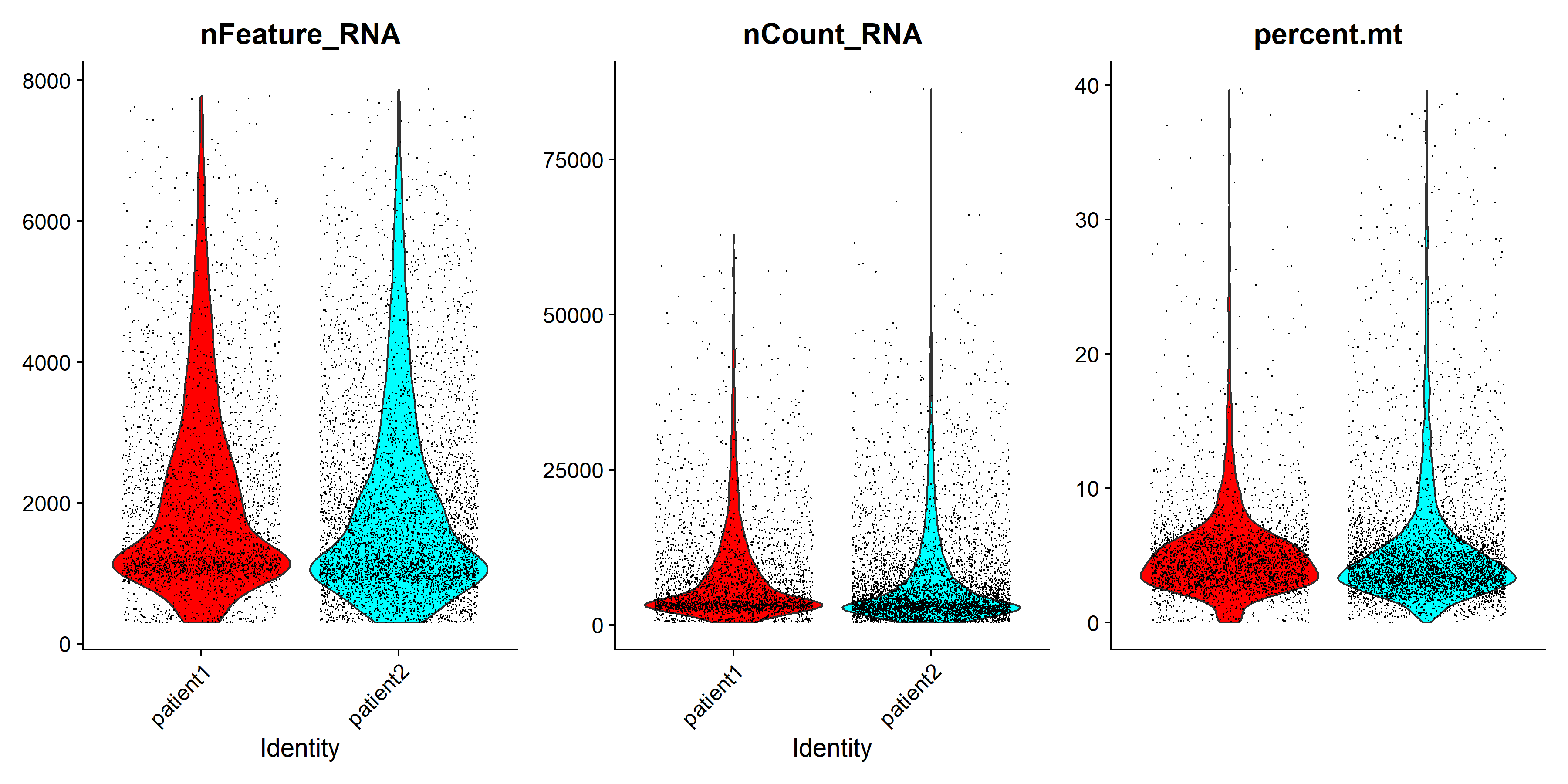

Supplement: Supplementary Tables 1 — The pyroptosis-related genes. [file DataSheet_1.zip › Supplementary FigureS4.jpg]

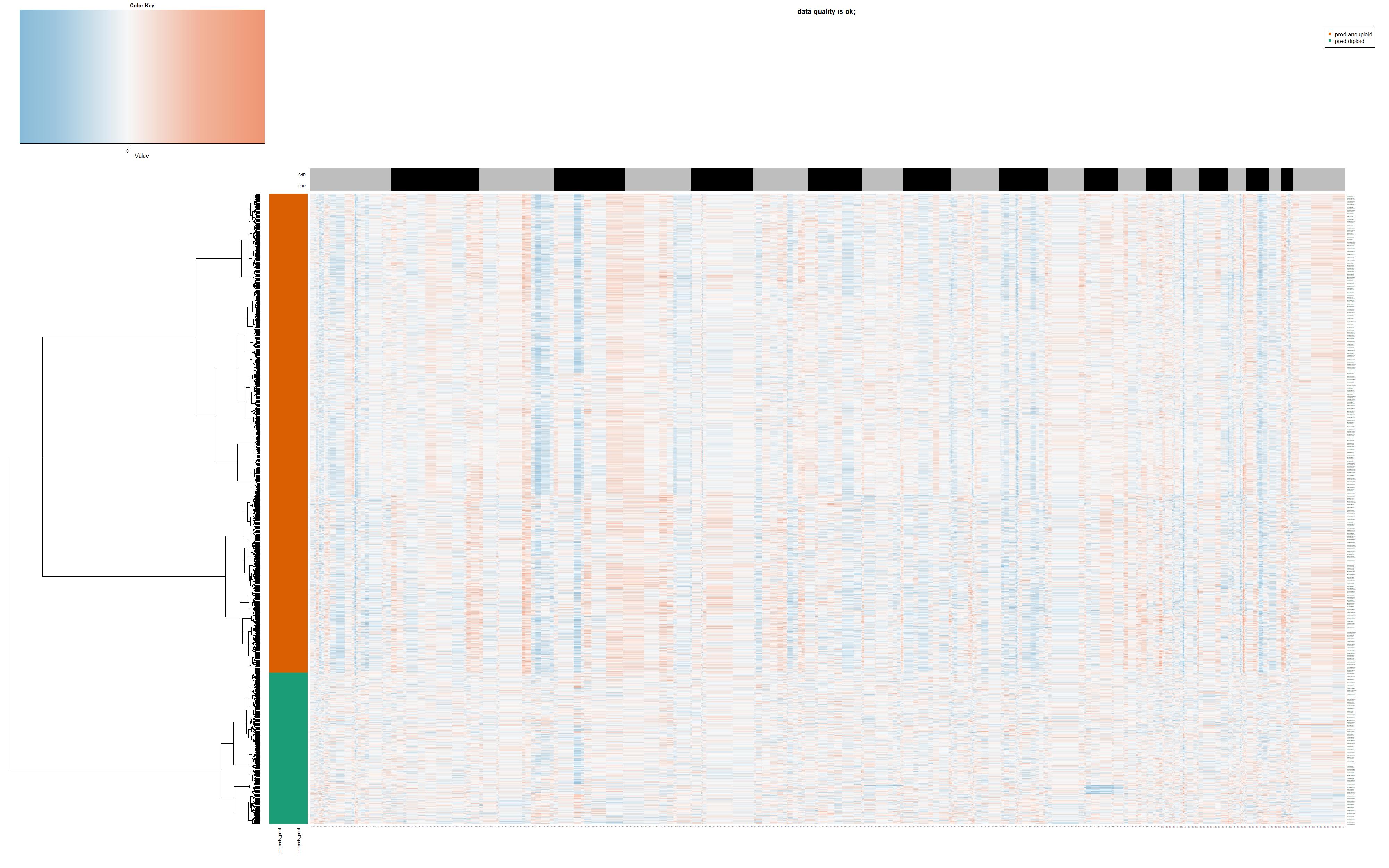

Supplement: Supplementary Tables 1 — The pyroptosis-related genes. [file DataSheet_1.zip › Supplementary FigureS5.jpg]

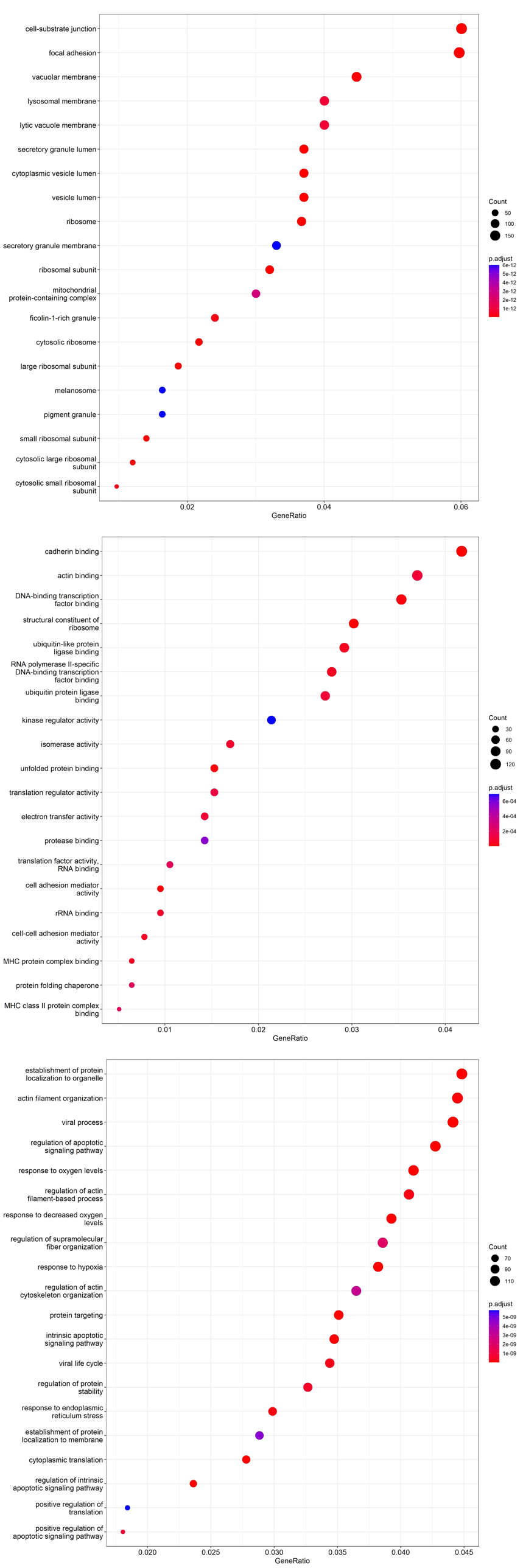

Supplement: Supplementary Tables 1 — The pyroptosis-related genes. [file DataSheet_1.zip › supplementary figuresS6.jpg]
